# Prominent Efficacy of Amantadine against Human Borna Disease Virus Infection In Vitro and In Vivo. Comment on Fink et al. Amantadine Inhibits SARS-CoV-2 In Vitro. *Viruses* 2021, *13*, 539

**DOI:** 10.3390/v14030494

**Published:** 2022-02-28

**Authors:** Liv Bode, Detlef E. Dietrich, Carsten W. Spannhuth, Hanns Ludwig

**Affiliations:** 1 Joint Senior Scientists, Freelance Bornavirus Workgroup, 14163 Berlin, Germany; hanns.ludwig@web.de; 2 Ameos Clinic Hildesheim Psychotherapy and Psychiatry, 31135 Hildesheim, Germany; detlef.dietrich@ameos.de; 3 Center for Systems Neuroscience, 30559 Hanover, Germany; 4 Department of Mental Health, Hannover Medical School, 30625 Hanover, Germany; cspann@gmx.net

**Keywords:** amantadine, human Borna disease virus, in vitro, in vivo, antiviral

## Abstract

Amantadine (1-amino-adamantane) is a versatile antiviral compound which has been licensed for decades against influenza viruses. During the Corona pandemic, its effect to inhibit SARS-CoV-2 in vitro has been investigated. However, an in vivo oral inapplicability was concluded due to ID_50_ doses exceeding eight times the estimated maximum tolerable plasma levels reached by 600 mg orally daily. In contrast, amantadine has been shown to be extraordinarily efficient against human neurotropic Borna disease virus (BoDV-1), presenting with both anti-depressive and anti-viral efficacy against a placebo, achieved by a well-tolerated low oral daily dose of 200 mg amantadine.

The SARS-CoV-2 pandemic has not only accelerated the development of novel vaccines against COVID-19 in record time—which have already saved millions of lives—but also the development of novel therapeutic drugs. On 22 December 2021, an emergency use authorization (EUA) for Pfizer’s Paxlovid was issued by the US Food and Drug Administration (FDA). Paxlovid (nirmatrelvir/ritonavir)—inhibiting the main protease, 3CL pro of SARS-CoV-2 [1]—reduced the proportion of people with COVID-19-related hospitalization or death by 88% compared to placebo within 28 days (NCT05011513).

Klaus Fink and colleagues published their in vitro study on amantadine against SARS-CoV-2 on 24 March 2021 [2], much earlier than the EUA for Paxlovid. Amantadine (1-amino-adamantane) is a well-known antiviral compound which was approved in 1966 by the FDA against influenza virus infection, but lost its recommendation by 2009 due to resistance against mutated viruses. Their rationale to evaluate its in vitro efficacy against SARS-CoV-2 was based on the hypothesis that the homo-pentameric ion channel of Corona E proteins could be a potential target. What they found was an ID_50_ reduction of SARS-CoV-2 by 83 µM amantadine in the supernatant and by 119 µM amantadine in infected Vero E6 cells. However, such drug concentrations would be not at all applicable in vivo, given that the highest tolerable in vivo dose of 600 mg—applied orally or intravenously—would lead to a plasma level of only 14.6 µM amantadine. Fink and colleagues argued that instead, a topical administration into the nose might be an alternate option, due to an achievable local level of 486 µM.

These results were disappointing with respect to SARS-CoV-2. Incomprehensible, however, was the lack of any information on recent data on amantadine’s superior efficacy against viruses other than influenza or SARS-CoV-2, namely Borna disease virus (BoDV-1). BoDV-1—a negative-strand RNA virus replicating in the nucleus and able to establish persistence in the brain and body—emerged as a potential human pathogen in the mid-1990s, when virus isolates could be achieved from psychiatric patients’ peripheral blood mononuclear cells (PBMCs) [3]. Thereafter, a startling discovery showed evidence for the efficacy of amantadine against human BoDV-1 strains in vitro and in vivo in a case report [4]. The link to psychiatric diseases initiated worldwide research but remained controversial. Recently, human encephalitis cases caused by BoDV-1 once again attracted public attention, although narrowing human BoDV-1 infection to only fatalities transmitted by shrews [5]. The more likely hypothesis of a wide array of diseases and human-to-human transmission goes against zoonosis; this is supported by worldwide prevalence data [6], but even more compellingly, by a double-blind placebo-controlled study on the efficacy of amantadine in depressed patients as well as in vitro [7]. The anti-depressive and antiviral effect could be achieved by a low and well-tolerated oral dose of 200 mg daily, which generated a plasma level of 1 µM amantadine (=0.4 µg/mL). In vitro, the ID_50_ dose of amantadine preventing infection with a human strain (BDV-Hu-H1) was achieved at 0.025 µM amantadine (=10 ng/mL). Notably, a closely related adamantane, memantine, failed up to a 100,000-fold higher concentration (500 µM; 200 µg/mL). The molecular target of amantadine for BoDV-1 still needs to be determined, but the specificity of amantadine compared to other adamantanes has been unequivocally demonstrated [7].

However, in terms of adamantanes, drug resistance could be a matter of concern. The well-tolerated dose of 200 mg amantadine daily—now shown to be effective against BoDV-1—had been successfully applied in the prophylaxis against influenza A virus infection [8] before an alarmingly high adamantane resistance rate of 92% abrogated any prophylactic use [9]. The main point mutation causing resistance was found to be a serine to asparagine change at amino acid 31 (S31N) of the M2 ion channel protein [9]. Whether the homo-pentameric E protein of SARS-CoV-2—which is also a channel protein (viroporin) [10]—would be less prone to adamantane resistance remained to be determined. In contrast, BoDV-1 strains have displayed an extremely high level of genetic conservation (>95% homology) [3,5], which may maintain a long-term sensitivity to amantadine rather than developing early resistance.

It is unfortunate that at least the double-blind study [7] has neither been discussed nor cited by Fink and colleagues [2]. The data are worth consideration given the pathogenic potential of human BoDV-1 infection.

## Data Availability

Not applicable.
